# USING COMMUNITY ENGAGEMENT TO DEVELOP, TEST, AND IMPLEMENT INTERVENTIONS FOR DEMENTIA CARE PARTNERS

**DOI:** 10.1093/geroni/igad104.0151

**Published:** 2023-12-21

**Authors:** Jacqueline Eaton

## Abstract

Interventions for caregivers to persons living with dementia are numerous and target a wide array of outcomes. One challenge limiting the dissemination of research-based interventions is the complexity of translating the intervention models for a variety of community environments and situations. Interventions that plan for future translation and scalability during the development stages have a better chance of quickly and efficiently being adopted by community partners. This symposium describes the methods used and the lessons learned from five research projects that used various forms of community engagement to develop, adapt, and assess feasibility of caregiver interventions, while simultaneously preparing for their translation and scalability: 1) Eaton et al., present the process and benefits gained from partnering with dementia caregivers to iteratively develop the Enhancing Active Caregiver Training (EnACT) intervention. 2) Utz et al., discuss ways to use the RE-AIM implementation framework to guide the development and pilot-testing of the Time for Living and Caring (TLC) respite intervention. 3) Dassel et al., explore the use of community-based participatory methods to adapt a paper-based advance care planning guide to an interactive web-based LEAD (Life-Planning in Early Alzheimer’s and other Dementias) intervention. 4) Cotton et al., report on the lessons learned from a formative analysis of their processes used to culturally adapt an intergenerational respite program for older adults within the Latine community. 5) Supiano et al., describe how they engaged and trained nursing home social workers to develop and implement the PreLoss Group Support intervention for dementia family care partners.

